# Birth “outside of guidance”—An exploration of a Birth Choices Clinic in the United Kingdom

**DOI:** 10.1111/birt.12827

**Published:** 2024-05-23

**Authors:** Sophie McAllister, Claire Litchfield

**Affiliations:** ^1^ Consultant Midwife at Oxford University Hospitals NHS Foundation Trust Oxford UK; ^2^ Consultant Midwife Trainee at Oxford University Hospitals NHS Foundation Trust Oxford UK

**Keywords:** birthplace, choice, guidelines, midwifery, personalized care

## Abstract

**Background:**

Decision‐making around birthplace is complex and multifactorial. The role of clinicians is to provide unbiased, evidence‐based information to support women and birthing people to make decisions based on what matters to them. Some decisions may fall outside of clinical guidance and recommendations. Birth Choices Clinics can provide an opportunity for extended discussion and personalized birthplace planning.

This study aimed to explore the rationale behind choosing birthplace “outside of guidance” and examine the outcomes for women who attended a Birth Choices Clinic.

**Methods:**

The study was descriptive using data extracted from clinical documentation and consultation. The data included demographic information, maternal characteristics, reason for choosing a midwifery‐led birth setting, birthplace preference, and outcome.

**Results:**

Eighty‐two women used the Birth Choices Clinic between April 2022 and February 2023 in one large maternity unit in the UK. Reasons for choosing birth in a midwifery‐led setting included having access to a birthing pool, to reduce the chance of obstetric interventions and pragmatic reasons. Sixty‐five percent of women experienced a spontaneous vaginal birth, 10% experienced an assisted vaginal birth, and 23% experienced a cesarean birth. Of the 33 women who ultimately commenced labor care in a midwifery‐led setting, 76% (*n* = 25/33) birthed in this setting without complications. Transfer rates in labor were similar to those in a “low‐risk” pregnant population.

**Discussion:**

Birth choice clinics may facilitate an understanding of material risk and support individualizing birth planning. There is evidence that women changed their planned birthplace, possibly in recognition of a move along the risk spectrum.

## 1 INTRODUCTION

In the United Kingdom, women and birthing people are encouraged to make choices about their maternity care, including place and mode of birth.^1^, ^2^ Choice and personalized care have been key themes highlighted by recent national reviews into maternity services.^3^, ^4^ Midwives have an important role in providing evidence‐based information to facilitate decision‐making and in being respectful of an individual's rights to accept or decline.^5^ Although women can employ independent midwives in the UK, the majority of maternity care is provided by National Health Service (NHS) organizations and employees.^6^

Recommendations about birthplace and fetal monitoring may be made by clinicians based on risk factors, which are often listed as inclusion or exclusion criteria for birth settings within clinical guidelines. In the UK, national guidelines are formulated by the National Institute for Care Excellence (NICE), which informs guidance at a local level.^7^ Clinical guidelines are important in providing guidance to clinicians that is based on the best available evidence to inform their practice. However, if used as a set of rules rather than guidance, guidelines can encourage a prescriptive approach to care at the expense of an individual's autonomy.^8^ Care needs to be taken to ensure “guideline‐centered care” does not supersede “person‐centered care,” and that clinicians work in partnership with women to ensure that opting for birth “outside of guidance” is not a seen as “disobeying” guidelines and viewed as deviant.^7^, ^8^

Choices that fall outside of recommended care could be described as “alternative,” “non‐normative,” “unconventional,” or “outside of guidance”.^7^, ^9^ This may be at either end of a spectrum, from seeking increased medical interventions, such as a planned cesarean, to planning to birth without medical assistance (known as freebirth). Here we focus on “alternative physiological birth choices” defined as:

*“Birth choices that go outside of local/national maternity guidelines or when women decline recommended treatment of care, in the pursuit of physiological birth.”* ^10^

Examples of physiological birth choices include choosing to birth in midwifery‐led settings with medical or obstetric risk factors, for example, planning birth at home after a previous cesarean, or choosing to decline routine practices such as fetal monitoring during labor.

This area of practice could present personal challenges for practitioners, both in facilitating antenatal discussions and in attending births. Supporting women choosing birth “outside of guidance” requires recognition of a person's right to respect for private and family life and the right to physical autonomy, which is enshrined in UK law.^10^, ^11^ A caregivers response to alternative choices can influence women's experiences of care, sometimes leading to women feeling coerced into complying with guidelines and affecting the relationship between the woman and clinician.^12^

Evidence suggests that midwives are, in principle, supportive of maternal autonomy^13^, ^14^ but may experience emotional distress and conflicting tensions when person declines care,^13^, ^15^ especially if these choices may increase the chance of complications.^10^, ^15^ Midwives have reported fear of poor birth outcomes or of professional accountability for poor outcome, with thorough documentation and personalized care plans developed by senior clinicians cited as the main way of managing tensions and reducing stress.^10^, ^13^, ^15^ Some NHS organizations offer Birth Choices Clinics to facilitate this senior review, supporting both midwives and clinicians. Little is currently known about the prevalence or operation of these clinics across the UK.

Decision‐making around birth is complex and influenced by service provision (e.g., what is physically available, staff knowledge or beliefs, and what information is available), and the beliefs, previous experiences, and preferences of women. In addition, the social and political climate and wider attitudes toward “natural birth”, risk, and “safety” may have an influence.^16^

Our exploratory descriptive study aims to contribute to knowledge about Birth Choices Clinics (BCC) and alternative physiological birth options, about which currently little is known.

## 2 METHODS

### 2.1 Study design and aim

This was a descriptive study evaluating the BCC, which aimed to describe the maternal characteristics of women attending BCC who wished to birth in midwifery‐led settings, identify common reasons for referral to BCC, and identify the rationale for opting to birth “outside of guidance”. We aimed to describe the frequency of change in planned birthplace after BCC consultation, and factors that may affect a change in preferred or planned birthplace and actual birthplace. We aimed to describe adverse maternal and perinatal outcomes, mode of birth, and transfers to an obstetric unit (OU) during labor or shortly after birth for those women attending the clinic.

### 2.2 Ethics

This study was a service evaluation. Approved by the NHS Trust Institutional Board Ethics approval ID 8271.

### 2.3 Birth Choices Clinic

The BCC operates within an NHS organization that is responsible for an obstetric unit (OU), an alongside midwifery unit (AMU), four freestanding midwifery units (FMU), and a home birth service. The BCC is run by consultant midwives and a consultant obstetrician and accepts referrals from clinicians working within the organization where women wish to explore their birth choices. During consultations, women are provided with information, clinical guidance, and rationale for recommendations. A detailed history taking of previous experiences and discussion of material risk (i.e., what is important to them) is used. A detailed birth plan is made with women making alternative physiological birth choices, designed to maximize safety in a setting that is not recommended. These plans are shared with women and those providing intrapartum care in advance so assurance can be provided that they have the support of the organization, and there is clear communication of discussions and plans.

Participants attended BCC between April 1, 2022 and February 28, 2023, and were referred as they wished to birth in a midwifery‐led setting where they had a characteristic that meant that they were not “included” in the eligibility criteria for home births or midwifery‐led units (MLU).

### 2.4 Data collection, management, and sources

Data were either collected from the maternal clinical record, from the referral form to clinic, or during the clinical consultation. A bespoke data collection tool using Microsoft Xcel was designed. The data were anonymized and held in a password protected file accessible to the authors.

#### 2.4.1 Data from maternal record

Data collected in the tool included maternal demographics (age, ethnicity, and parity), which were available from the maternal clinical record. This included the planned birthplace at the start of labor, whether this had changed since the BCC appointment, and if so, the reason for change was collected. Data about transfer during labor to an OU and any reason for this was collected, as well as data about the actual birthplace. The maternal record was also the source for data on outcomes, including mode of birth and any adverse maternal or neonatal events.

#### 2.4.2 Data from the referral form

Data about the reason women had been advised to birth in an OU were collected iteratively upon receipt of the referral and described in a free text box. These could include maternal factors, current pregnancy‐related factors, or previous pregnancy factors.

#### 2.4.3 Data from clinical consultation

Data were collected about planned birthplace before and after the BCC appointment, and included options of home, AMU, FMU, and OU. Data about the rationale for the decision to birth in a midwifery‐led setting was collected by the clinic team. This rationale was explored and discussed during the clinic appointment and recorded in a free text box.

### 2.5 Data analysis

Maternal demographics were described using frequencies and percentages. Data about planned birthplace, reason for change in birthplace, reason for any transfer, and actual birthplace were described using frequencies and percentages.

Textual data describing reasons women were “outside of guidance” were grouped into categories and described using frequencies and percentages.

Textual data describing the rationale for choosing birthplace “outside of guidance” was grouped into descriptive themes, and a narrative descriptive analysis of these themes was performed.

Where uncommonly serious adverse outcomes occurred, all available data, including free text, were reviewed to understand the circumstances surrounding these events.

## 3 RESULTS

### 3.1 Ethnicity, age and parity

Eighty‐two women attended the BCC and were included in the study.

The characteristics are described in Table 1. Most women were from a white ethnic background (84.2%/*n* = 69), 11.0% (*n* = 9) were from a Black, Asian, or mixed ethnicity background, and 4.9% (*n* = 4) of participants ethnicity was unknown. This was compared with the local population using maternity services; people from a global ethnic majority (GEM) background were underrepresented (17.2% of women are from GEM communities in the wider population, compared with 11% in the study population).

**TABLE 1:** Characteristics of women attending Birth Choices Clinic requesting birth outside of guidance.

	*n* (%)
Maternal age (years)	
21–25	4 (4.9%)
26–30	26 (31.7%)
31–35	27 (32.9%)
36–40	23 (28.1%)
41–45	2 (2.4%)
Ethnicity	
Asian or any other Asian—Background	1 (1.2%)
Asian or Asian British—Indian	3 (3.7%)
Asian or Asian British—Pakistani	2 (2.4%)
Black or Black British—African	2 (2.4%)
Mixed—any other mixed	1 (1.2%)
Other—not stated	4 (4.9%)
White—any other white background	8 (9.8%)
White British	60 (73.2%)
White Irish	1 (1.2%)
Parity	
0	11 (13.4%)
1	34 (41.5%)
2	19 (23.2%)
3	3 (3.7%)
4	8 (9.8%)
5	2 (2.4%)
6	5 (6.1%)

Most women were aged under 35 (69%), and the majority were under 40 years old (92.7%) (*n* = 76/82).

Seventy‐one participants were multiparous (one or more previous birth), and 11 were primiparous (no previous births) (86.6% vs. 13.4%). Fifteen were “grand multiparous” (4 or more previous births).

### 3.2 Factors for which OU birth is recommended

Table 2 outlines the risk factors, which meant participants were defined as “higher risk” and therefore birth in an OU was recommended. The most common factors were previous postpartum hemorrhage (PPH) (20), previous cesarean (19), and grandmultiparity (14).

**TABLE 2:** Factors for which OU birth is recommended.

Maternal factors	(*n*)	Current pregnancy‐related factors	(*n*)	Previous pregnancy factors	(*n*)
BMI > 35	10	42 weeks gestation or more	2	4 or more previous births	14
BMI < 18	1	Anemia	3	Previous cesarean birth	19
Hypertension/preeclampsia	2	Polyhydramnios	1	Previous shoulder dystocia	2
Gestational diabetes (diet‐controlled)	3	Raised uterine artery dopplers >3	8	Previous postpartum hemorrhage	20
Gestational diabetes (medicated)	1	Low PAPP‐A < 0.3MoM	1		
Other maternal medical condition (e.g., asthma)	3	Other (thrombocytopenia, group B strep, fetal abnormality)	3		
Fibroids	1				

Ninety‐six risk factors were identified among the 82 participants. Sixty‐eight women had 1 risk factor (83.9%), and 14 women had 2 or more risk factors in their pregnancy identified (17.1%).

For multiparous women, the most common risk factors were previous cesarean, grandmultiparity and previous PPH. For primiparous women, a raised BMI >35 was the most common risk factor, which excluded them from planning birth at home or in a FMU, according to local guidance.

### 3.3 Motivation for considering place of birth “outside of guidance”

Eighty women with obstetric or medical risk factors were considering birth in a midwifery‐led setting, and two women without any risk factors were declining elements of recommended monitoring in labor.

Four main themes were identified:

- The desire to experience **water immersion** using a birth pool during labor.
- **Pragmatic factors,** which meant birth at home or in a MLU, were more practical. This included the MLU being closer to home and logistically easier to organize childcare for older children. Some women reported previous rapid births and they were concerned they would not reach the OU in time to give birth and may even birth in transit.
- **Experience of birth**: Women felt the chosen birth setting was more likely to lead to a positive, calm, or more comfortable birth experience. Sometimes this was due to a previous positive experience of birth at home or in an MLU, or due to a poor or traumatic previous experience of birth in an OU.
- **Reduction in obstetric interventions**: Women wanted to reduce their risk of experiencing obstetric interventions such as unplanned cesareans or birth with forceps or ventouse and increase their chance of having a physiological birth.

### 3.4 Place of birth

**FIGURE 1:**
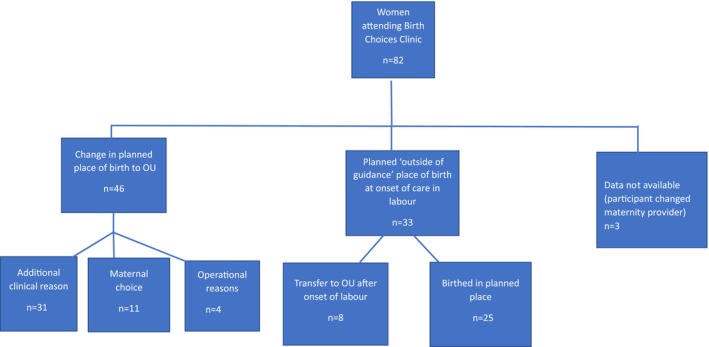
Participants journey after attending Birth Choices Clinic. [Colour figure can be viewed at wileyonlinelibrary.com]

Figure 1 describes the journey of participants to their final birthplace. Thirty‐three participants were planning birth “outside of guidance” at the onset of care in labor. Data on planned birthplace at the start of labor and transfer was unknown for 3 participants (3.7%) as they changed maternity practitioners and were lost to follow‐up.

Forty‐six participants (56.1%) either changed their planned birthplace to the OU in pregnancy before the start of care in labor (91.3%, *n* = 42), or were unable to birth at home or in a MLU due to operational issues such as lack of available staff or no capacity at the chosen location (8.7%, *n* = 4).

The most common reasons for change in planned birthplace were a change in the clinical situation (67.4%, *n* = 31), including induction or stimulation of labor (45.7%, *n* = 21), maternal decision in pregnancy (23.9%, *n* = 11), prolonged rupture of membranes (8.7%, *n* = 4), requesting an epidural (4.4%, *n* = 2), preterm birth (4.4%, *n* = 2), hypertension (2.2%, *n* = 1), and fetal malpresentation (2.2%, *n* = 1).

Of the 82 participants, 50 gave birth on an OU (61%), 16 gave birth at home (19.5%), 6 gave birth in an AMU (7.3%), 7 gave birth in a FMU (8.5%), and 2 gave birth with a different maternity practitioner, whose birthplace was unknown.

### 3.5 Transfers to an obstetric unit during labor or immediately postpartum

Thirty‐three women (see Figure 1) received care at home or on an MLU in labor; 75.7% (*n* = 25/33) birthed in this setting with no complications. Eight women (24.2%) transferred to an OU during labor or immediately after birth (3/8 transferred from an AMU, 2/8 transferred from an FMU, and 3/8 transferred from home). Two were primiparous, and six were multiparous women. However, two of the multiparous women had previous cesarean and no previous vaginal births. Reasons for transfer are outlined in Table 3.

**TABLE 3:** Indication for transfer to the obstetric unit.

Delay in first stage of labor	1
Delay in second stage of labor	1
Meconium‐stained liquor	2
Neonatal condition at birth (i.e., low o2 sats, resuscitation needed)^a^	2
Non‐urgent transfer post birth for routine neonatal observations	1
Suspected fetal compromise/death	1

^a^ Upon transfer, reviewed by neonatal team but no further treatment and not admitted to the Neonatal Unit.

### 3.6 Mode of birth

Fifty‐three participants had a spontaneous vaginal birth (64.7%), 19 women had a cesarean (23.2%), and 8 women had a birth with forceps or ventouse (9.7%).

Table 4 reports the mode of birth in the study population overall and by location at the onset of care in labor. A higher proportion of women had a spontaneous vaginal birth who were in a midwifery‐led setting at the onset of care in labor than those in an OU (88% compared with 52%). A lower proportion of women had an unplanned cesarean who were in a midwifery‐led setting at the onset of care in labor than those in an OU (3% compared with 26%).

**TABLE 4:** Mode of birth.

Mode of birth	All (*n* = 82)	Home/MLU at onset of care in labor (*n* = 33)	OU at onset of care in labor (*n* = 46)
Birth with forceps	7.3% (*n* = 6)	9% (*n* = 3)	7% (*n* = 3)
Birth with ventouse	2.4% (*n* = 2)	0	4% (*n* = 2)
Planned cesarean birth	6.1% (*n* = 5)	0	11% (*n* = 5)
SVB	64.7% (*n* = 53)	88% (*n* = 29)	52% (*n* = 24)
Unknown	2.4% (*n* = 2)	*	*
Unplanned cesarean birth	17.1% (*n* = 14)	3% (*n* = 1)	26% (*n* = 12)
		* *N* = 3	

* Changed maternity provider therefore outcome unknown.

### 3.7 Maternal and perinatal outcomes

#### 3.7.1 Adverse outcomes

Overall, there were 13/82 adverse events (15.9%), 80.4% (*n* = 66) of women experienced no adverse event, and 3.66% (*n* = 3) were unknown as lost to follow up. These events were admission to the neonatal unit (*n* = 2), term intrauterine death (IUD) diagnosed at the onset of labor care (*n* = 1), PPH (blood loss 500–1500 mL) (*n* = 10), and uterine rupture diagnosed at cesarean (*n* = 1).

## 4 DISCUSSION

The study suggests there is a demand for birth “outside of guidance” and provides insight into the reasons why women choose midwifery‐led settings when not recommended. Women who are “higher risk” wish to reduce their chances of obstetric intervention, experience water immersion, and believe that midwifery‐led settings will provide them with a more positive birth experience. Women choosing birth at home or in a MLU close to where they lived had legitimate practical concerns about travel time to the OU and their ability to arrange childcare for older children.

Midwives and doctors must practice evidence‐based care, ensuring recommendations are based on the best available evidence.^5^ In 2015, a landmark legal case changed care in the UK, with the addition that clinicians must ensure the birthing person is aware of any material risks involved in the recommended treatment or plan of care. A “material” risk is one that a reasonable person in the woman's position would likely attach significance to, or if the clinician is or should reasonably be aware that the woman would likely attach significance to it.^17^

Therefore, clinicians must listen to and understand their patients, considering what is important to an individual, which will vary from person to person. Our study has revealed a group of women whose choices are sometimes motivated by need rather than personal preference. Clinical consultations need to explore the women's personal situation, as we have found this can affect their “choices.” For example, women who have previously birthed quickly and therefore birth in an OU, although recommended, may not be realistic or achievable.

The desire to use water immersion in labor was the most common reason for choosing birth at home or in an MLU. This demonstrates that women value this resource highly and supports the importance of birth pools being available on OUs so that women with pregnancy complications may also have access to water immersion in labor. During the study period, the birth pool on the OU was not in use. This is likely to have increased the number of higher‐risk women choosing to birth in a midwifery‐led setting, as this would have been the only way to access a birth pool in labor. This finding is supported by literature that has explored women's experience of using water immersion as positive,^18^ while facing multiple barriers to access.^19^

Women described choosing a birth setting that provided a positive experience of birth and reduced the chance of obstetric interventions. Evidence suggests that women are more likely to have a spontaneous vaginal birth if birth is planned in a midwifery‐led setting.^20^, ^21^, ^22^ This group of women were motivated by birth experience and physiological birth and were making choices that would optimize their chance of this. For some women, this was due to a previous traumatic birth experience in an OU and highlights the profound affect that this may have on future birth planning.

After the BCC appointment, just over half of women (51.2%) changed their planned birthplace, either due to a change in their preference or in the clinical situation. This is an interesting finding and warrants further investigation, in particular the effect of BCC on decision‐making and, where plans have changed due to operational reasons, what effect this may have on future birth planning. It may be, for example, that this reduces confidence in the care provider.

Of those who commenced labor in a midwifery‐led setting as planned, the majority remained in this setting and birthed without complications. Transfer rates in labor or immediately after birth (24.2%) were similar or lower to those found in other studies, although we cannot directly compare as we have not accounted for possible confounding factors.

Previous studies suggest women with uncomplicated pregnancies having their first baby have a 36%–45% chance of transfer, and those having second or subsequent births have a 9%–13% chance of transfer to OU during labor or immediately postpartum.^20^, ^23^ A study of women with a previous cesarean who planned birth at home found transfer rates were 37% and varied widely by parity.^21^

There are differences in transfer rates for women who have had a previous vaginal birth compared with those who had not. The numbers in this study were too small to allow for a subgroup analysis of transfer rates by previous vaginal birth or by a specific risk factor.

The number of spontaneous vaginal births was higher in this study compared with the general population at the maternity unit in 2022 (65% vs. 45%).^24^ Our study has similar findings to the BirthPlace Study,^20^ with a higher proportion of women who attended BCC achieving vaginal birth if their labor care at the onset was in a midwifery‐led setting.

While there were some adverse outcomes, the risk profile of the women was high, and the majority of women experienced vaginal birth with no complications. Most adverse events were experienced by women who had changed their planned birthplace during pregnancy and attended the OU at the onset of labor (*n* = 10/13).

We propose that there is a natural process of “filtering” of women either, away from, or toward birthplace “outside of guidance”, depending on the accumulation of additional risk factors.

For example, women with a raised BMI may have an otherwise uncomplicated pregnancy, and others may develop preeclampsia or gestational diabetes later in pregnancy, which means they move away from planning birth in a midwifery‐led setting. Perhaps this can be attributed to the power of a having a pregnancy complication diagnosed rather than being counseled about an increased *chance* of developing a complication that has not actually occurred. Where a pregnancy complication is diagnosed, clinicians may make stronger recommendations or counsel more persuasively.

The “filtering” described may suggest that women who birth “outside of guidance” in a midwifery‐led setting are at the lower end of a risk spectrum, than those who recognize the accumulation of multiple risk factors and change planned birthplace because of this. Low risk or high risk is often discussed as a binary concept to categorize pregnancies to decide on pathways of care. We suggest this is an unhelpful concept which groups women into 1 of 2 groups. Considering risk as a spectrum on which a birthing person can move along may more accurately describe the reality of the complexity of individuals.

While we focus on the UK experience, there may be benefit in offering a BCC in other maternity settings, particularly where maternity care practitioners may be financially incentivized to provide particular mode/place of birth.

### 4.1 Strengths and limitations

The study design was a service evaluation; designed and conducted to define the current service, with the aim of improving the service for women and clinicians. However, this design and the small heterogenous sample mean the findings are not generalizable.

Our evaluation is exploratory and descriptive in nature intended to inform future research on this topic where currently little is known. We recognize the limitations of our approach, particularly with the qualitative data, and caution must be exercised in interpreting the results.

### 4.2 Conclusion and recommendations

Birth is a biopsychosocial‐cultural event,^7^ and women make birth choices based on a wide range of factors, depending on what is important to them. Clinicians have a duty to understand what the material risk is for the individual.

We suggest that women may recognize a move along the risk spectrum and adjust their birthplace plans accordingly.

BCCs can offer the opportunity to maximize safety by developing and communicating clear plans rather than reiterating recommendations that may not be achievable or realistic. We recommend sharing the philosophy of the BCC with women to avoid assumptions that it represents an additional barrier to their choice.

Future studies could explore the rationale for and effect of changes in the planned birthplace for those wishing to choose alternative physiological birth options.

Evaluation of women's experience of attending Birth Choices Clinics, and midwives' experiences of following Birth Choices Clinic plans for women who choose a birthplace “outside of guidance,” should be explored in future studies.

## CONFLICT OF INTEREST STATEMENT

CL and SM have no conflicts of interest to declare.

## Data Availability

The data that support the findings of this study are available from the corresponding author upon reasonable request.
